# A comparative analysis of multi-level computer-assisted decision making systems for traumatic injuries

**DOI:** 10.1186/1472-6947-9-2

**Published:** 2009-01-14

**Authors:** Soo-Yeon Ji, Rebecca Smith, Toan Huynh, Kayvan Najarian

**Affiliations:** 1 Department of Computer Science, Virginia Commonwealth University, 401 East Main Street, Richmond, Virginia, USA; 2 Department of Surgery, Carolinas Healthcare System, Charlotte, NC, USA

## Abstract

**Background:**

This paper focuses on the creation of a predictive computer-assisted decision making system for traumatic injury using machine learning algorithms. Trauma experts must make several difficult decisions based on a large number of patient attributes, usually in a short period of time. The aim is to compare the existing machine learning methods available for medical informatics, and develop reliable, rule-based computer-assisted decision-making systems that provide recommendations for the course of treatment for new patients, based on previously seen cases in trauma databases. Datasets of traumatic brain injury (TBI) patients are used to train and test the decision making algorithm. The work is also applicable to patients with traumatic pelvic injuries.

**Methods:**

Decision-making rules are created by processing patterns discovered in the datasets, using machine learning techniques. More specifically, CART and C4.5 are used, as they provide grammatical expressions of knowledge extracted by applying logical operations to the available features. The resulting rule sets are tested against other machine learning methods, including AdaBoost and SVM. The rule creation algorithm is applied to multiple datasets, both with and without prior filtering to discover significant variables. This filtering is performed via logistic regression prior to the rule discovery process.

**Results:**

For survival prediction using all variables, CART outperformed the other machine learning methods. When using only significant variables, neural networks performed best. A reliable rule-base was generated using combined C4.5/CART. The average predictive rule performance was 82% when using all variables, and approximately 84% when using significant variables only. The average performance of the combined C4.5 and CART system using significant variables was 89.7% in predicting the exact outcome (home or rehabilitation), and 93.1% in predicting the ICU length of stay for airlifted TBI patients.

**Conclusion:**

This study creates an efficient computer-aided rule-based system that can be employed in decision making in TBI cases. The rule-bases apply methods that combine CART and C4.5 with logistic regression to improve rule performance and quality. For final outcome prediction for TBI cases, the resulting rule-bases outperform systems that utilize all available variables.

## Background

According to a 2001 National Vital Statistics Report [1], nearly 115,200 deaths occur each year due to traumatic injury, and many patients who survive suffer life-long disabilities. Among all causes of death and permanent disability, traumatic brain injury (TBI) is the most prevalent. Of the 29,000 children who are hospitalized each year with TBI, a significant percentage will suffer from neurological impairment [2]. It has also been reported that the traumatic brain injuries are the most expensive affliction in the United States, with an estimated cost of $224 billion [3].

Computer-aided systems can significantly improve trauma decision making and resource allocation. Since trauma injuries have specific causes, all with established methods of treatment, fatal complications and long-term disabilities can be reduced by making less subjective and more accurate decisions in trauma units [4]. In addition, it has been suggested that an inclusive trauma system with an emphasis on computer-aided resource utilization and decision making may significantly reduce the cost of trauma care [1].

Since the treatment of traumatic brain injuries is extremely time-sensitive, optimal and prompt decisions during the course of treatment can increase the likelihood of patient survival [5,6]. It is also believed that the predicted length of stay in the ICU is an important factor when deciding on the patient transport method (i.e. ambulance or helicopter), as more critical patients are expected to spend more time in the ICU, and these stand to benefit the most from helicopter transport. Studies have emphasized the critical impact of helicopter transport on trauma mortality rates, since the speed of ambulance transport is limited by road and weather conditions, and may also be constrained by traffic congestion. However, it is difficult to compare ground and helicopter transportation and the corresponding care provided to the patients [7]. Cunningham [8] attempts a comparison based on the outcome of the treatment given to trauma patients. Based on his study, patients in critical condition are more likely to survive if transported via helicopter. However, the high cost of helicopter transport remains a major problem [9,10]. In recent studies, Gearhart evaluated the cost-effectiveness of helicopter for trauma patients and suggested that on average the helicopter transport cost is about $2,214 per patient, and $15,883 for each additional survivor [11]. Eventually, the cost is almost $61,000 per surviving trauma patient. Eckstein [12] states that 33% of patients who are transported by helicopter are discharged home from the emergency department [12], rather than being sent to ICU. This indicates that a significant number of trauma patients transported by helicopter actually have relatively minor injuries. This emphasizes the necessity of a comprehensive transport policy based on patient condition and predicted outcome.

Several computer-assisted systems already exist for decision-making in trauma medicine. The majority of these systems [13,14] are designed to perform a statistical survey of similar cases in trauma databases, based only on patient demographics. As such, they may not be sufficiently accurate and/or specific for practical implementation. Other medical decision making systems employ the predictive capabilities of artificial neural networks [15-17]; however, due to the 'black box' nature of these systems, the reasoning behind the predictions and recommended decisions is obscured. Currently, none of these existing systems are in widespread use in trauma centers. There are three main reasons: the use of non-transparent methods, such as neural networks; the lack of a comprehensive database integrating all relevant available patient information for specific prediction processes; and poor performance due to the exclusion of relevant attributes and the inclusion of those irrelevant to the current task, resulting in rules that are too complicated to be clinically meaningful.

Several machine learning algorithms are commonly applied to medical applications. These include support vector machines (SVM), and decision tree algorithms such as Classification and Regression Trees (CART) and C4.5. Boosting is also employed for improving classification accuracy. However, despite the relatively successful performance of these algorithms in medical applications, they have limited success in separating and identifying important variables in applications where there are a large number of available attributes. This suggests that combining machine learning with a method to identify the most uncorrelated set of attributes can increase our understanding of the patterns in medical data and thus create more reliable rules. The literature of biomedical informatics reinforces the benefits of this approach. Andrews et al. [18] use decision tree (DT) and logistic regression (LR) methods to identify the commonalities and differences in medical database variables. Kuhnert [19] emphasises that non-parametric methods, such as CART and multivariate adaptive regression splines, can provide more informative models. Signorini et al. [20] design a simple model containing variables such as age and GCS, but the small number of attributes may limit the reliability of the generated rules. Guo [21] finds that CART is more effective when combined with the logistic model, and Hasford [22] compares CART and logistic regression, and finds that CART is more successful in outcome prediction than logistic regression alone.

Therefore, a possible approach to create accurate and reliable rules for decision making is to combine machine learning and statistical techniques [23,24]. This paper analyzes the performance of several combinations of machine learning algorithms and logistic regression, specifically in the extraction of significant variables and the generation of reliable predictions. Though a transparent rule-based system is preferable, other methods (such as neural networks) are also tested in the interest of comparision. A computational model is developed to predict final outcome (home or rehab and alive or dead) and ICU length of stay. In addition, we identify the factors and attributes that most affect decision making in the treatment of traumatic injury.

Our hypotheses are as follows:

1. We hypothesize that a rule-based system, attractive to physicians as the reasoning behind the rules is transparent and easy to understand, can be as accurate as "black-box" methods such as neural networks and SVM.

2. We hypothesize that when trained correctly, a computer-aided decision making system can provide clinically useful rules with a high degree of accuracy.

3. Studies mentioned earlier have examined which variables are most significant in the recommendation/prediction making process. We hypothesize that airway status, age, and pre-existing conditions such as myocardial infarction and coagulopathy are significant variables.

## Methods

Rules are created by processing patterns discovered in the traumatic brain injury (TBI) datasets. More specifically, they are generated by analyzing the logical and grammatical relationships among the input features and the resulting outcomes. Rules are formally defined as grammatical expressions of knowledge extracted using specific logical operations on the available features [6].

CART and C4.5 are among the most popular algorithms for creating reliable rules, but they are limited in their ability to identify the most significant variables. We therefore perform statistical analysis using logistic regression, which is typically effective in discovering statistically significant regression coefficients [24]. Although stepwise regression is designed to find significant variables, it may not perform well with CART when dealing with small scale datasets [25]. Therefore, in this paper, logistic regression with direct maximum likelihood estimation (Direct MLE) is used.

### Dataset

Three different datasets are used in the study: on-site, off-site, and helicopter. The on-site dataset contains data captured at the site of the accident; the off-site dataset is formed at the hospital after patients are admitted; and the helicopter dataset consists of the records for patients who are transported to hospital by helicopter. The on and off-site datasets are used to predict patient survival (dead/alive) and final outcome (home/rehab), and the helicopter dataset is used to predict ICU length of stay, which is a measure used in estimating the need for helicopter transportation. The datasets are provided to us by the Carolinas Healthcare System (CHS) and the National Trauma Data Bank (NTDB).

#### On-site dataset

When making decisions based on the variables available at the accident scene, one has to consider the unavailability of important factors such as pre-existing conditions (comorbidities). Decisions must therefore be made without knowledge of these factors. Some physiological measurements are also excluded because they are only collected after arrival at the hospital. Table 1 presents the variables collected for this dataset, which consist of four categorical and six numerical attributes.

**Table 1:** On-site dataset

Variable	Possible Values
Gender*	2 (Male, Female)

Blunt*	Blunt, Penetrating

ChiefComp*	MVC, Fall, Pedestrian, Motorcycle Crash, etc

Position*	Passenger, Driver, Cyclist, Motorcycle Passenger, etc

Age	Patient's age

FSBP (Initial Blood Pressure)	0 ≤ FSBP ≤ 300

GCS (Glasgow Coma Score)	3 ≤ GCS ≤ 15

ISS (Total Injury Severity Score)	0 ≤ ISS ≤ 75

Pulse	0 ≤ Pulse ≤ 230

Respiration Rate	0 ≤ Respiration ≤ 68

Categorical variables are starred.

#### Off-site dataset

The off-site dataset contains information on comorbidities and complications, and includes all variables. A total of 1589 cases are included in the database: 588 fatal and 1001 non-fatal. The inputs include both categorical and numerical attributes. The predicted outcomes are defined as the patients' survival, i.e. alive or dead, and the exact outcome for surviving patients, i.e. rehab or home.

Table 2 presents the variables for our dataset. Among the categorical variables, "prexcomor" represents any comorbidities that may negatively impact a patient's ability to recover from the injury and any complications. Other terms are defined in the table description.

**Table 2:** Off-site dataset

Variable	Alive	Dead	Rehab	Home
Cases	1001	588	628	213

Male*	704 (70.3%)	404 (68.7%)	443 (70.5%)	150 (70.4%)

Female*	297 (29.7%)	184 (31.3%)	185 (29.5%)	63 (29.6%)

Age	41.2 ± 19.6	49.2 ± 24.1	39.6 ± 19.3	37.2 ± 16.6

FSBP	126 ± 33.4	119.3 ± 45.6	125.3 ± 31.6	124.5 ± 34.1

FURR	15.3 ± 10.9	13.9 ± 11.9	14.4 ± 11.1	18.2 ± 10.5

GCS	8.7 ± 5.3	27.5 ± 5.2	7.9 ± 5.2	10.5 ± 5.1

ISS	30.5 ± 12.8	35.3 ± 14.7	32 ± 13.2	27.1 ± 11.7

EDEYE	2.4 ± 1.4	2.1 ± 1.4	2.2 ± 1.4	2.8 ± 1.4

ED Verbal	2.7 ± 1.8	2.3 ± 1.7	2.4 ± 1.8	3.3 ± 1.8

EDRT	4.6 ± 3.2	3.8 ± 3.3	4.1 ± 3.3	5.7 ± 2.89

Head AIS	3.0 ± 1.6	3.6 ± 1.6	3.1 ± 1.8	2.5 ± 1.4

Thorax AIS	2.3 ± 1.7	2.4 ± 1.8	2.3 ± 1.8	2.4 ± 1.7

Abdomen AIS	1.1 ± 1.5	1.1 ± 1.6	1.0 ± 1.5	1.5 ± 1.7

Intubation*	Yes/No			

Prexcomor*	17 values: Acquired Coagulopathy, Chronic Alcohol Abuse, Chronic Obstructive Pulmonary Disease, Congestive Heart Failure, Coronary Artery Disease, Coumadin Therapy, Documented History of Cirrhosis, Gastric or Esophageal Varices, Hypertension, Insulin Dependent, Myocardial Infarction, Non-Insulin Dependent, Obesity, Pre-existing Anemia, Routine Steroid Use, Serum Creatinine > 2 mg % (on Admission), Spinal Cord Injury			

Complications*	Acute Respiratory Distress Syndrome (ARDS), Aspiration Pneumonia, Bacteremia, Coagulopathy, Intra-Abdominal Abscess, Pneumonia, Pulmonary Embolus			

Safety*	Seat Belt, None Used, Air Bag Deployed, Helmet, Other, Infant/Child Car Seat, Protective Clothing			

First, the number of cases in each group (Alive, Dead – and within the surviving patients, Rehab and Home) is listed. For the numerical attributes, the table provides Mean ± Standard Deviation. Finally, the categorical variables are listed with all their possible values. ISS provides the overall injury severity score (ISS) for patients with multiple injuries, and GCS is the Glasgow Coma Score. Many studies make heavy use of GCS and ISS, as these measures are considered standard metrics in assessing patient condition and degree of injury. Note that surviving patients who were transported to other hospitals are not included in the rehab + home total.

#### Helicopter dataset

This dataset is formed based on the records of patients who were transported to hospital by helicopter. The variables are age, gender, blood pressure, cheifcomp (the type of injury), airway (the type of device used to assist patients with breathing), prefluids (the amount of blood provided to the patients), GCS, heart rate, respiration rate, ISS-Head&Neck, and ISS. Age, blood pressure, GCS, heart rate, ISS-Head&Neck, ISS, and respiration rate are classified as numerical variables. The final outcome is the number of days spent in ICU, as this is considered the most informative measure when deciding the means of transport to hospital. In our dataset, ICU stay ranges between 0 and 49 days. The use of a relatively small dataset with so many outcomes may result in a complex model that is hard to explain and understand. Inspired by Pfahringer [26], the dataset is classified into two groups. The non-severe group contains patients who stayed in the ICU less than 2 days (ICU stay ≤ 2 days). The severe group consists of patients who stayed in the ICU more than 2 days (ICU stay ≥ 3 days). This threshold was chosen based on discussion with trauma experts. In total, the dataset contains 497 cases: 196 severe and 301 non-severe [10]. Table 3 describes the helicopter dataset in more detail.

**Table 3:** Helicopter dataset

Variable	Severe (ICU stay > 2 days)	Non-Severe (ICU stay ≤ 2 days)
Cases	301	196

Male	201 (66.8%)	132 (67.3%)

Female	100 (33.2%)	64 (32.7%)

Age	30.6 ± 16.6	32.9 ± 17.2

FSBP	137.7 ± 23.2	127.6 ± 28.0

GCS	11.7 ± 4.87	6.47 ± 5.01

ISS	14.2 ± 8.1	23.7 ± 9.47

Pulse	101.4 ± 22.3	108.2 ± 26.6

Resp. Rate	15.6 ± 9.44	6.45 ± 10.6

ISS-HN	2.83 ± 0.86	3.46 ± 0.91

The number of severe and non-severe cases is listed, along with the percentages of each that are male and female. Mean ± standard deviation is given for each numerical attribute.

### Learning algorithms

It is known that the patterns observed in trauma cases are often extremely complicated; that is, the treatment outcomes for two apparently similar patients may turn out to be significantly different. Linear methods have proven insufficient even in the analysis of patterns as simple as the "exclusive-or" function. Because these limitations are inherited by linear regression methods, the use of non-linear techniques for computer-aided trauma systems has been broadly encouraged [27]. Neural networks are a common choice; however, they are not transparent, since the knowledge learned from the training examples is hidden within the structure and weights of the networks [28]. While there are existing methods that can extract approximate rules to represent this hidden knowledge, they cannot truly represent the trained networks [6]. Support Vector Machines (SVM's) and AdaBoost share the same problem: the knowledge used in the decision making process is not visible to humans, a requirement that is extremely important in medical applications. Rule-based methods such as CART and C4.5 provide completely transparent computational decision making systems while still utilizing some nonlinear capabilities. Considering the importance of decision transparency in medical informatics, we use CART and C4.5 as the main algorithms for rule extraction.

#### Classification and Regression Tree (CART)

CART, designed by L. Breiman [29], applies information-theoretic concepts to create a decision tree. This allows for the capture of rather complex patterns in data, and their expression in the form of transparent grammatical rules [30]. CART's nonlinear extensions are still widely used in data mining and machine learning, due to the algorithm's efficiency in dealing with multiple data types [31] and missing data. In the latter case, CART simply uses a substitution value, defined as a pattern similar to the best split value in the node [29]. In addition, CART supports an exhaustive search of all variables and split values to find the optimal splitting rules for each node. The splitting stops at the pure node containing fewest examples.

#### C4.5

C4.5 [15,32,33] extends Quinlan's basic ID3 decision tree algorithm [34]. It is more successful in avoiding overfitting, is able to handle continuous variables, and is more computationally efficient. To generate rules, C4.5 uses a divide-and-conquer algorithm to split training data into disjoint regions of the variable space, according to pre-assigned target labels [9]. At each step, C4.5 splits on the best attribute according to the gain criterion. This criterion is based on entropy, i.e. the randomness of the class distribution in the dataset. The criterion is the greatest difference in entropy of the class probability distribution of the current subset S and the subsets generated by the split.

(1) $$Info(S)=-\sum_{i=1}^{n}p(k_{i},S)\cdot \log_{2}p(k_{i},S)$$

where *p*(*k*_*i*_, *S*) is the relative frequency of examples in S that belong to class *k*_*i*_. The best split is the one that most reduces this value. The output of the algorithm is a decision tree, which can be easily represented as a set of symbolic IF-THEN rules.

#### Adaptive Boost (AdaBoost)

AdaBoost, introduced by Freund and Schapire [35], is an algorithm that constructs a robust classifier as a linear combination of weak classifiers. Adaboost repeatedly calls a given weak learning algorithm in a set of rounds *t *= 1, ..., *T*. A distribution of weights is maintained over the training set, such that *D*_*t*_(*k*) is the distribution's weight for training example k on round t. The aim of the weak learner is to find a good weak hypothesis *h*_*t*_: *X *→ {-1, +1} for the distribution *D*_*t*_, where goodness is measured by the error of the hypothesis with respect to *D*_*t*_. Then *D*_*t *_is updated such that incorrectly classified examples have their weights increased, forcing the weak classifier to concentrate on the more difficult training examples. Correspondingly, correctly classified examples are given less weight. Adaboost selects some parameter *α*_*t *_to denote the importance of *h*_*t*_, and after all rounds are complete, the final hypothesis *H *is a weighted majority vote of all *T *weak hypotheses. It has been shown that as with other boosting algorithms, if each weak hypothesis is at least slightly better than random, then the training error falls at an exponential rate. However, Adaboost is also able to adapt to the error rates of individual weak hypotheses, so each subsequent classifier is adjusted in favor of examples mislabelled by previous classifiers [36].

#### Support Vector Machine (SVM)

SVMs [37] are supervised learning methods used primarily for classification. An SVM treats its input data as two sets of vectors in n-dimensional space: positive and negative examples. In this space, it constructs an optimal hyperplane that preserves the maximum distance between the two sets [38]. Since SVM is able to handle large feature spaces, it has been successfully used in solving many real world problems such as text categorization, image classification, protein analysis, cancer data classification, and hand-writing recognition [39]. Consider a set of N labelled training examples *D *= (*x*_1_, *y*_1_),..., (*x*_*n*_, *y*_*n*_) with *y*_*i *_∈ {+1, -1} and *x *∈ *R*^*d*^, where *d *is the dimensionality of the input. Let *φ*: *R*^*d *^→ *F *be the mapping function from the input space to the feature space. If the two classes are linearly separable, the SVM algorithm finds a hyperplane (*w*, *b*) that maximizes the margin

(2) $$\gamma =\underset{i}{\min}\{y_{i}<w,\phi (x_{i})>-b\}$$

where *b *is a real number (bias term) and *w *and *F *have the same dimensionality. For an unknown input vector *x*_*j*_, classification means finding:

(3) *f*(*x*_*j*_) = *sgn*(*y*_*i *_<*w*, *φ*(*x*_*i*_) > -*b*)

It can be shown that this minimum occurs when *w *= Σ_*i*_*α*_*i*_*γ*_*i*_*φ*(*x*_*i*_), where *α*_*i *_is a positive real number that represents the strength of training point *x*_*i *_in the final classification decision. The subset of points where ai is non-zero consists of the points closest to the hyperplane, and these are the support vectors. Since SVM is able to handle large feature spaces, it is frequently used in many real world problems even though it is computationally expensive [39].

#### Neural networks

A neural network processes training examples individually, and learns by comparing its classification of the input (which is initially largely arbitrary) with the given correct classification. In particular, Radial Basis Function (RBF) networks are well suited to solving pattern classification problems due to their simple topological structure and their capability for faster learning. A standard RBF network is a supervised feed-forward back propagation neural network, consisting of an input layer, a hidden layer and an output layer. One of the most common basis functions for the hidden layer is the family of Gaussian functions whose outputs are inversely proportional to the distance from the center of the neuron. Given a finite set of training data {(*x*_*j*_, *y*_*j*_)|*j *= 1, . . ., *m*}, and the center vector of basis function *c*_*i*_, the equation for a simple output is:

(4) $$y_{j}=\varphi (x)=\sum_{i=1}^{N}\alpha_{i}\rho (||x_{j}-c_{i}||)$$

where *N *is the number of neurons in the hidden layer, and *α*_*i *_are the weights minimizing least square between real output and approximate output. Typically a Gaussian activation function producing a radial function of the distance between each hidden unit weight vector and each pattern vector is used as a basis function:

(5) $$\rho (||x_{i}-c_{i}||)=\exp(-\frac{\left||x_{i}-c_{i}|\right|}{\sigma^{2}})$$

where *σ *indicates the neuron radius [40,41]. RBFs utilize the distance in feature space to calculate the weight for each neuron.

### Pre-processing

The datasets contain nominal categorical variables, such as gender and complication type. Gender is replaced by a binary variable (0 for male, 1 for female). Every nominal value is dummy-coded (Yes/No to 1/0) and treated as an individual attribute. Ten fold cross-validation is used to measure the generalization quality and scalability of the rules. Each dataset is divided into ten mutually exclusive subsets [42], and in each stage nine are used for training and one is used for testing. Ten different trees are therefore formed for each dataset.

### Rule performance metrics

Once a variety of rules are generated, the performance of each rule is measured as the probability of correct prediction. Assume that *D *is a dataset including the instance (*x*_*i*_, *y*_*i*_), where *y*_*i *_is the real survival outcome. Let *D*_*r *_be the training set, and a subset *D*_*t *_∈ (*D*\*D*_*r*_) be used for testing. The performance of the rule is calculated as:

(6) *acc*_*R *_= *prob*(*y*_*i *_= *y*^*R*^|(*x*_*i*_, *y*_*i*_) ∈ *D*_*t*_)

where *y*^*R *^is the outcome produced by induction, i.e. the expected classification. The number of positive matches in the testing set is used as a measure of rule accuracy. Rule accuracy can also be estimated as follows:

(7) $$Accuracy=\frac{TP+TN}{TP+TN+FP+FN}$$

where TP is the number of true positives, TN is the number of true negatives, FP is the number of false positives, and FN is the number of false negatives. Sensitivity and specificity are then used to assess the quality of the rules. These measures are useful, as they calculate the probabilities of false positives and false negatives separately; one may be significantly higher than the other, and this can be obscured in a single average error measure. The formulae for these measures are shown below.

(8) $$Sensitivity=\frac{TP}{TP+FN}$$

(9) $$Specificity=\frac{TN}{FP+TN}$$

where TP, TN, FP, and FN are defined as before. In this application, high sensitivity is more important than high specificity. When patient lives are at stake – for example, in the choice of transportation – false positives are preferable to false negatives, even if they incur greater financial cost.

### Improving rule quality

Once the most accurate rules have been extracted, direct maximum likelihood estimation with logistic regression is used to improve rule quality. The logistic function calculates the expected probability of a dichotomy as:

(10) $$\pi_{i}=pr(Y=1|X)=\frac{1}{1+e^{-(\beta_{0}+\beta_{1}X_{1}+\beta_{2}X_{2}+...)}}$$

where *X*_*i *_are variables with numeric values, *Y *is the outcome (dichotomous; 0 or 1, e.g. Alive/Dead), and the *β*'s are the regression coefficients that quantify the contributions of the numeric variables to the overall probability [22].

Logistic regression provides knowledge of the relationships and strengths among the multiple independent variables and the response variable. It does not assume any distribution on the independent variables; they do not have to be normally distributed, linearly related or of equal variance within each group. The most important interpretation from logistic regression is the odds ratio, which measures the strength of the partial relationship between an individual predictor and the outcome event [43].

**Figure 1:**
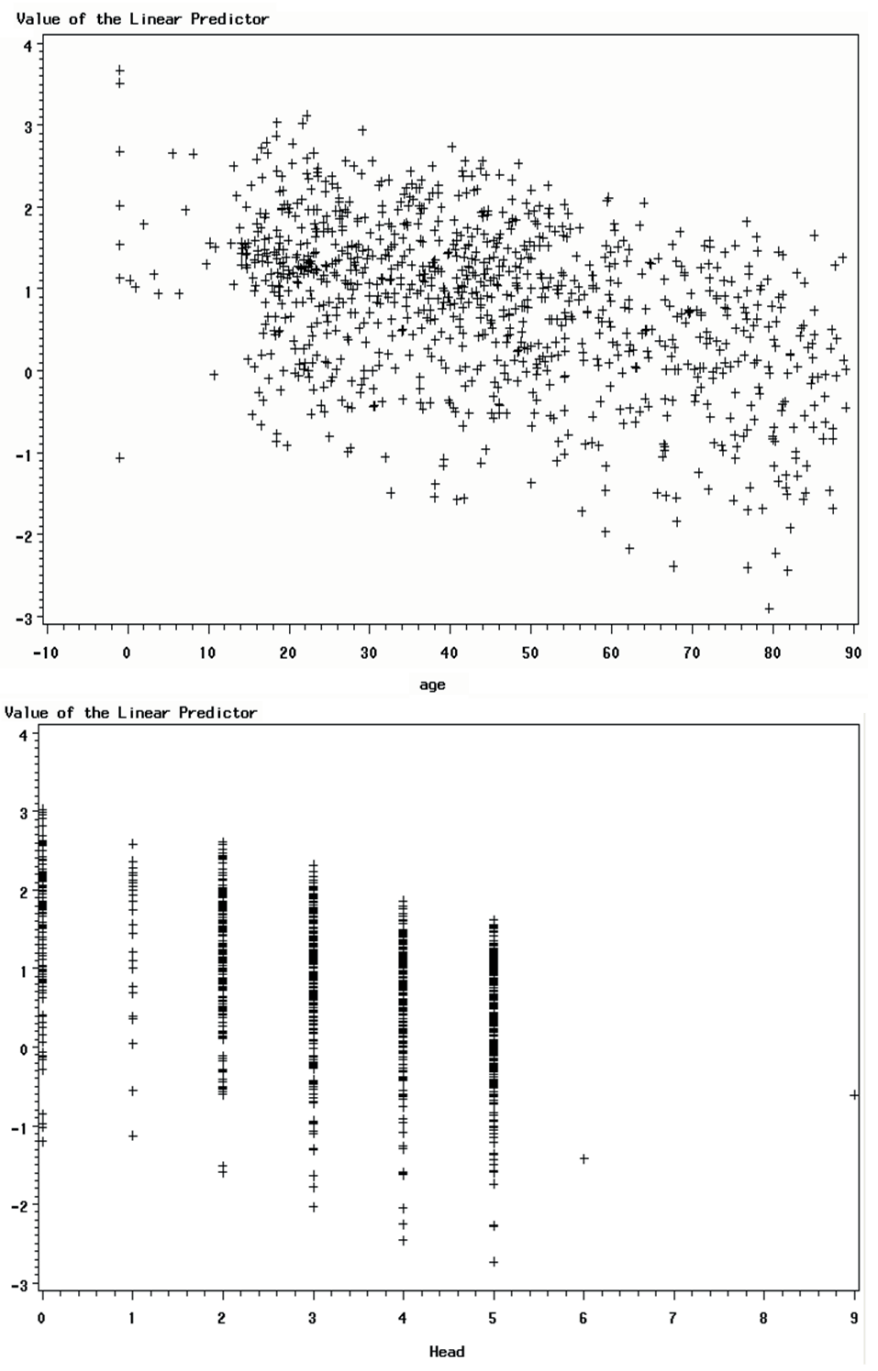
**Scatter plots of logits and predictors**. This figure presents two scatter plots, used to demonstrate that the relationship between the logit and the predictors is linear. The first scatter plot is of logit vs. Age (a continuous variable), and the second is of logit vs Head AIS (a discrete variable).

**Figure 2:**
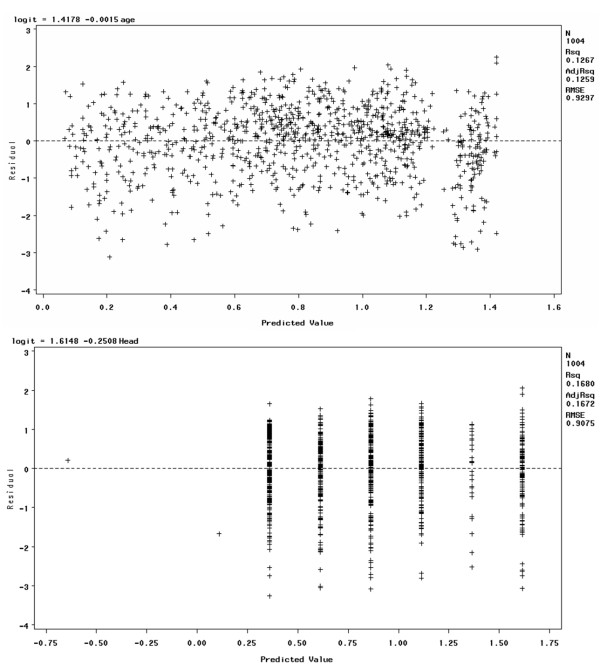
**Residual plots for logits and predictors**. This figure presents two residual plots, used to demonstrate that the relationship between the logit and the predictors is linear. These plots were made using regression analysis. The first residual plot is between logit and Age (a continuous variable), and the second is between logit and Head AIS (a discrete variable).

The advantage of using the logit scale for interpretation is that the relationship between the logit and the predictors is linear. To check this linearity assumption, we used scatter plot and residual analysis. The results showed linear relationships for all variables, though some were weaker than others. In the interests of brevity, in this paper we present the results for only two variables: Head AIS and Age. First, we present the scatter plot between the logit and its predictor, and then the residual plot between them using regression analysis. If the linearity assumption is satisfied, we would expect the residuals to vary randomly – i.e. they would not demonstrate any pattern. If the residual plot appears to form a curve, there may be a nonlinear relationship in the variable. This analysis was performed using statistical analysis software (SAS). Figure 1 and Figure 2 present the scatter plots and residual plots using Age and Head AIS as the predictors for patient survival.

If the plots of the residuals versus the predictors do show curvature, a quadratic term should be tested for statistical significance for suggesting better model. If the coefficient for this quadratic term is significant, the quadratic term should be included. Even though our model does not show any strong curvature, we test the Head AIS variable using a quadratic term, to validate our results. The model is as follows:

(11) *logit *= *α *+ *βx *+ *γx*^2^

where *α *is an intercept term, *β *is a parameter of the predictor, and *γ *is a parameter of squared predictor. For the Head AIS variable, the estimate of *β *is -0.1820 (p value = 0.0015), and the estimate of *γ *is -0.0124 (p value = 0.2058). These p values indicate that Head AIS does not require a quadratic term; therefore, there is a linear relationship between the logit and its predictor.

To test the significance of the individual variables, we compare a reduced model that drops one of the independent variables with a full model using log likelihood test. The likelihood ratio test itself does not tell us if any particular independent variables are more important than others. However, by estimating the maximum likelihood, we can analyze the difference between results for the full model and results for a nested reduced model which drops one of the independent variables. A non-significant difference indicates no effect on performance of the model, hence we can justify dropping the given variable. We call this directed MLE.

The test takes the ratio of the maximized value of the likelihood function for the full model (*L*_1_) over the maximized value of the likelihood function for the simpler model (*L*_0_). The resulting likelihood ratio is given by:

(12) $$-2\log(\frac{L_{0}}{L_{1}})=-2[\log(L_{0})-\log(L_{1})]=-2(L_{0}-L_{1})$$

If the chi-square value for this test is significant, the variable is considered to be a significant predictor. Following these tests, only the significant variables (p value <= .05) are selected.

Note that forward and stepwise model selections are also available to discover the significance of individual attributes [19,25]. In the literature of statistical regression, the stepwise method is commonly used to find the best subset of variables for outcome prediction, considering all possible combinations of variables. However, the stepwise approach may not guarantee that the most significant variables are selected due to the repetition of insertion and deletion. For example, age may not be selected as important variable; however, physicians may believe that patient age is important in deciding treatment. Therefore, we prefer to use directed MLE for our medical application. Our other justification for using MLE is empirical; in our previous study [10], we found that the direct MLE method has slightly higher accuracy in finding significant variables than stepwise and forward model selection. A statistical analysis tool, in this case SAS, is used to calculate the significance of individual attributes.

### Constructing reliable rules

As mentioned previously, SVM and neural networks do not directly produce grammatical rules; therefore, only CART and C4.5 are considered for rule extraction. Those variables identified as significant are used as input variables to CART and C4.5. Also, if a rule is created only to accommodate one or two examples, it may be too specific to be applied to the entire population. Consequently, only the rules with both high accuracy and a sufficiently large number of supporting examples are used to form the rule base. Note that SVM, Neural Networks and AdaBoost are still tested in the interests of performance comparision, even though they do not generate rules. These algorithms are in widespread use, and comparing them to the rule based CART and C4.5 algorithms tests and validates the accuracy and stability of the rule-based system.

## Results

The average accuracy of survival prediction without any knowledge of pre-existing conditions is 73.9%, rising to 75.8% when this knowledge is included. The off-site dataset is therefore used for further prediction tests, as it contains records of pre-existing conditions. We discovered that knowledge of these conditions appears at the highest level of the tree when using CART and C4.5, indicating their potential importance in the decision-making process. In particular, coagulopathy (bleeding disorder), which can result in severe haemorrhage, may be among the most important factors to consider in patients with TBI.

Due to the transparent nature of the rule-based system used in this study, the generated rules can not only help trauma experts predict the likelihood of survival, but also provide the reasoning behind these predictions in order to help physicians better allocate their resources.

Since the total number of examples used for training is rather small, initially only rules with at least 85% prediction accuracy on the testing sets are included in the rule base. This threshold was chosen following discussion with trauma experts. However, we also incorporate rules with accuracy between 75% and 85%. There are two reasons for this. Firstly, the accuracy of a rule may be low due to the lack of of a truly complete database, rather than a flaw in the rule itself. Secondly, even though a rule may have low accuracy, it might include knowledge of hidden relationships between variables. For example, most trauma experts consulted believed that a patient with an ISS score over 25 would have little chance of survival. However, the survival probability might be higher for a patient with a high ISS score, but lower head and thorax AIS score, provided appropriate and prompt treatment is provided. Therefore, we will use those rules with accuracy between 75% and 85% as additional "supporting rules" in suggesting possible treatment. This issue is addressed further in the discussion section.

### Significant variable selection

In order to improve the rule quality and accuracy, it is essential that we identify the key variables in the dataset. In addition, shorter rules that are based on fewer, more significant variables are more clinically useful for physicians. Direct MLE with logistic regression is used to accurately extract these key variables from our helicopter and off-site datasets; the results for the off-site dataset are presented in Table 4. It can be seen that nine important variables are identified. Using standard deviations, Wald chi-squares are computed on each variable and the odd ratios are interpreted as showing a strong relationship between the outcome and the independent variables. Table 5 presents the significant variables extracted from the helicopter dataset. Only five of the eleven original variables are identified as significant.

**Table 4:** Significant variables of off-site dataset

Variable	Coefficient	**Walds χ**^2^	P-value	Odd Ratios	Mean ± S.D.
AIS Head	-0.58	23.61	<.0001	0.56	3.25 ± 1.64

AIS Thorax	-0.13	4.37	0.003	0.88	2.33 ± 1.78

ID*	1.27	5.70	0.02	3.55	-

MI*	1.43	19.44	<.0001	4.18	-

ARDS*	0.98	20.24	<.0001	2.66	-

Cg*	0.63	24.96	<.0001	1.88	-

Age	-0.03	29.22	<.0001	1.03	44.15 ± 21.70

EDRTS	-0.27	4.94	0.03	0.77	12.10 ± 16.03

ISS	0.02	6.06	0.01	1.02	15.82 ± 19.03

Categorical variables are starred. Cg stands for Coagulapathy; MI for Myocardial Infarction; ARDS for Acute Respiratory Distress Syndrome; ID for Insulin Dependent; EDRTS for Emergency Department Revised Trauma Score; ISS for Injury Severity Score.

**Table 5:** Significant Variables of Helicopter dataset

Variable	Coefficient	**Wals χ**^2^	P-value	Odd Ratios	Mean ± S.D.
Age	-0.02	3.17	<.0001	0.98	31.79 ± 17.50

Blood Pressure	0.01	2.85	0.01	0.01	129.45 ± 30.51

ISS-HN	0.01	0.003	0.25	1.11	3.22 ± 1.00

ISS	-0.14	36.47	0.02	0.87	19.56 ± 11.09

ISS stands for Injury Severity Score; ISS-HN for Head/Neck Injury Severity Score.

In this study the scale of the data is small and several variables are unknown, so participating physicians assisted in identifying significant variables. These physicians selected age, GCS, blood pressure, pulse rate, respiration rate, and airway as important factors.

### Measuring performance

The prediction results of five different machine learning methods are compared in Table 6. The performance for all algorithms is clearly superior when only significant variables are used. In addition, using only the most significant variables is shown to result in a more balanced testing-training performance. Discussion with physicians revealed that generated recommendations and predictions must be transparent in their reasoning; our system therefore uses CART and C4.5 to predict patient survival. If physicians understand the reasoning behind decisions and it follows their own, their confidence in the system may be increased. If the system's reasoning is clinically meaningless, they can choose to disregard the recommendation; however, if the reasoning has some clinical merit, this may alert them to previously hidden factors affecting patient outcome.

**Table 6:** Performance comparison of five machine learning methods

	Logistic	AdaBoost	C4.5	CART	SVM	RBF NN
All Variables	69.4%	70%	68%	75.6%	73%	67.2%

Significant Vars. only	72.9%	73%	75.2%	77.6%	79%	79.04%

The five chosen machine learning algorithms are AdaBoost, C4.5, CART, SVM, and RBF Neural Network.

Table 7 presents the performance accuracy in outcome prediction (rehabilitation or home) for the off-site dataset, and prediction of ICU days for the helicopter dataset. In both cases, only the significant variables are used. No attempt is made to use all available variables, since the survival prediction test has already confirmed the improved performance when using only significant variables.

**Table 7:** Prediction results for outcome and ICU days

	Logistic	AdaBoost	C4.5	CART	SVM	RBF NN
Exact Outcome	74.6%	73%	75.6%	72%	72.6%	72.8%

Days in ICU	80.6%	78.7%	77.1%	77.4%	80.1%	77.4%

This table compares the performance of logistic regression alone and the five chosen machine learning algorithms in predicting exact outcome (for off-site dataset) and ICU length of stay (for helicopter dataset).

**Figure 3:**
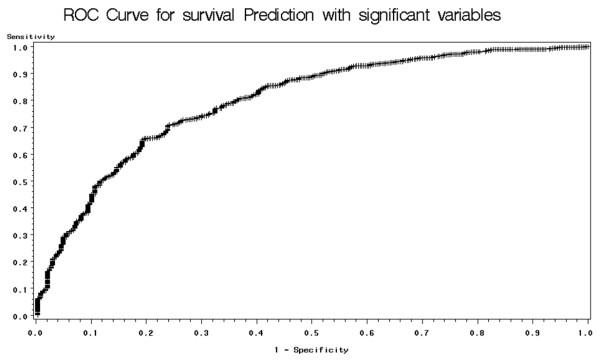
**ROC plot for Logistic regression on survival prediction**. This figure presents the ROC plot obtained when applying logistic regression for survival prediction, using only significant variables. Tables 8 and 9 contain AUC (area under curve) results for the other machine learning methods and the other prediction scenarios.

**Figure 4:**
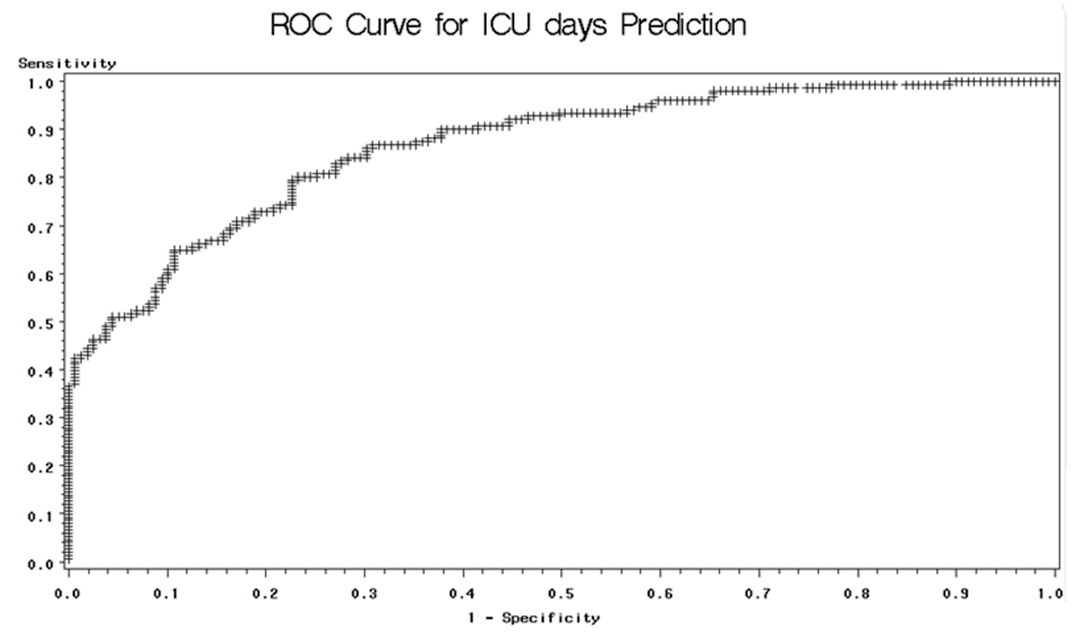
**ROC plot for Logistic regression on ICU days prediction**. This figure presents the ROC plot obtained when applying logistic regression for ICU days prediction, using only significant variables.

We also generate Receiver Operating Characteristic (ROC) curves – plots of the true positive rate (sensitivity) versus the false positive rate (1-specificity) – in order to evaluate the model performance. First, we perform ROC analysis on the patient surivival prediction results. Table 8 compares the area under the curve (AUC) for the ROC curves generated using all available variables and significant-only variables. The table shows that results are improved when using only significant variables in the model. Therefore, when dealing with the helicopter dataset, we only perform ROC analysis on the significant-variable-only model. The results are presented in Table 9. Notice that there is no large difference in ROC analysis results among the various machine learning methods. However, when the dataset is small – such as our data used for ICU days prediction – logistic regression outperforms the other methods. Figure 3 and Figure 4 present sample ROC plots for logistic regression using only significant variables for survival and ICU days prediction respectively.

**Table 8:** Performance comparison of AUC in ROC curve analysis

	Logistic	AdaBoost	C4.5	CART	SVM
All Variables	63.7%	63.1%	58.1%	60%	64.5%

Significant Vars. only	66.9%	67.5%	63.2%	64.6%	67.6%

**Table 9:** ROC performance in Exact outcome and ICU days predictions

Variable	Logistic	AdaBoost	C4.5	CART	SVM
Exact outcome	76.8%	76.4%	71.9%	71.5%	68.7%

Days in ICU	79.2%	74.6%	76.6%	73%	71.9%

### Constructed database using CART and C4.5

Numerous rules were generated with the CART and C4.5 rule extraction algorithm. Following discussion with trauma experts, we identified the robust rules as those with over 85% accuracy. For survival prediction, the average rule accuracy using all available variables is 82%, and 83.9% when using only the most significant variables.

Table 10 presents the most reliable generated rules for survival prediction (> 85% accuracy); Table 11 contains survival rules with accuracy between 75% and 85%. Similarly, Table 12 presents the most reliable generated rules for outcome prediction (> 85% accuracy), and Table 13 contains outcome rules with accuracy between 75% and 85%. Finally, Table 14 presents the most reliable generated rules for ICU days prediction (> 85% accuracy), and Table 15 contains ICU days rules with accuracy between 75% and 85%. Note that the rules with accuracy between 75% and 85% may not be sufficiently reliable, yet may contain useful pattern information, as described in the discussion section.

**Table 10:** Extracted reliable rules for survival prediction (> 85% accuracy)

Rules	Test Accuracy	Method
(Cg = 'Yes') and HEAD < 2 and AGE < 76.65 Then Alive	29/34(85.3%)	CART

(Cg = 'No') and (MI = 'No') and AGE < 61.70 and HEAD ≤ 4 and (ARDS = 'No') Then Alive	334/375(89.1%)	CART

(Cg = 'No') and (MI = 'No') and HEAD ≥ 5 and AGE < 22.35 Then Alive	55/64(85.9%)	CART

ISS ≥ 28 and (Cg = 'No') and THORAX ≤ 4 and 62.25 ≤ AGE < 69.00 and EDRTS ≥ 2.88 Then Alive	10/11(90.9%)	CART

ISS ≥ 23 and (Cg = 'No') and THORAX ≤ 4 and 69 ≤ AGE < 72.35 Then Alive	13/15(86.7%)	CART

HEAD ≤ 2 and (MI = 'No') and (Cg = 'No') and AGE ≤ 62 Then Alive	182/206(88.3%)	C4.5

(MI = 'Yes') and AGE ≤ 62 and EDRTS > 5.39 and ISS ≤ 25 Then Alive	19/20(95%)	C4.5

THORAX > 3 and HEAD ≤ 4 and (ARDS = 'No') and AGE ≤ 62 Then Alive	126/148(85.1%)	C4.5

THORAX ≤ 2 and EDRTS ≤ 0.87 and ISS > 38 Then Dead	12/13(92.3%)	C4.5

(MI = 'Yes') and AGE > 82.6 Then Dead	16/18(88.9%)	C4.5

(MI = 'Yes') and ISS > 30 Then Dead	45/50(90%)	C4.5

HEAD > 4 and (MI = 'Yes') Then Dead	25/27(92.6%)	C4.5

(Cg = 'Yes') and HEAD ≤ 4 and AGE > 78 Then Dead	12/14(85.7%)	C4.5

(ID = 'Yes') and AGE > 78 and (MI = 'Yes') and HEAD ≤ 4 Then Dead	27/31(87.1%)	C4.5

HEAD > 0 and HEAD ≤ 2 and (ID = 'Yes') and (ARDS = 'No') and AGE ≤ 75.2 Then Alive	107/118(90.7%)	C4.5

(ID = 'Yes') and (MI = 'Yes') and HEAD > 3 Then Dead	43/49(87.8%)	C4.5

(MI = 'Yes') and (ID = 'Yes') and AGE > 78 Then Dead	32/37(86.5%)	C4.5

HEAD > 4 and (MI = 'Yes') Then Dead	25/27(92.6%)	C4.5

(MI = 'Yes') and ISS > 30 Then Dead	45/50(90%)	C4.5

(MI = 'Yes') and AGE > 79.6 and ISS > 12 Then Dead	27/30(90%)	C4.5

(Cg = 'Yes') and HEAD ≤ 4 and AGE > 79.6 Then Dead	12/14(85.7%)	C4.5

(ARDS = 'No') and (MI = 'No') and (Cg = 'No') and HEAD ≤ 4 and AGE ≤ 62 Then Alive	335/376(89.1%)	C4.5

(MI = 'Yes') and (ID = 'Yes') and AGE > 78 Then Dead	15/16(93.8%)	C4.5

(MI = 'Yes') and HEAD ≤ 4 and ISS > 38 Then Dead	29/34(85.3%)	C4.5

(MI = 'Yes') and AGE ≤ 61.6 and ISS > 27 Then Dead	26/30(86.7%)	C4.5

HEAD = 2 and (MI = 'No') and AGE ≤ 62 and ISS ≤ 38 Then Alive	235/270(87%)	C4.5

THORAX > 0 and (ID = 'Yes') and ISS ≤ 30 Then Alive	13/14(92.9%)	C4.5

Reliable rules are defined as those with accuracy greater than 85%. Cg stands for coagulopathy; MI for myocardial infarction; ARDS for Acute Respiratory Distress Syndrome; EDRTS for Emergency Department Revised Trauma Score; ISS for Injury Severity Score; ID for Insulin-Dependent.

**Table 11:** Extracted supporting rules for survival prediction (75% – 85% accuracy)

Rules	Test Acc.	Method
(Cg = 'Yes') and 2.5 ≤ HEAD < 3.5 and EDRTS < 6.07 and 35.65 ≤ AGE < 55.25 Then Alive	10/12(83.3%)	CART

(Cg = 'Yes') and HEAD ≥ 3 and EDRTS ≥ 6.07 and THORAX < 1 Then Alive	33/43 (76.7%)	CART

(Cg = 'No') and (MI = 'No') and AGE < 61.70 and (ARDS = 'Yes') and HEAD < 3 Then Alive	50/59(84.7%)	CART

(Cg = 'No') and (MI = 'No') and ISS = 24 and 61.70 = AGE < 68.90 and HEAD ≤ 3 Then Dead	11/13(84.6%)	CART

AGE < 61.70 and HEAD ≤ 4 and (MI = 'No') Then Alive	625/793(78.8%)	CART

HEAD ≥ 5 and (Cg = 'No') and AGE < 22.85 Then Alive	60/73(82.2%)	CART

HEAD ≥ 5 and (Cg = 'No') and EDRTS < 5.02 and 22.85 ≤ AGE < 28 and ISS ≥ 33 Then Dead	11/13(84.6%)	CART

ISS ≥ 23 and (ID = 'Yes') and 61.70 ≤ AGE < 80.50 and (ARDS = 'No') and (Cg = 'Yes') Then Dead	12/15(80.0%)	CART

ISS ≥ 23 and (ID = 'Yes') and AGE ≥ 80.50 Then Dead	42/51(82.4%)	CART

AGE < 61.70 and (Cg = 'Yes') and HEAD ≤ 3 and ISS < 42 Then Alive	47/56(83.9%)	CART

AGE < 61.70 and (Cg = 'No') and (MI = 'No') Then Alive	559/706 (79.2%)	CART

(MI = 'No') and (Cg = 'Yes') and HEAD ≤ 3 and AGE < 60.40 and ISS < 42 Then Alive	47/56(83.9%)	CART

(MI = 'No') and (Cg = 'No') and ISS ≤ 23 and EDRTS < 6.07 Then Alive	578/728(79.4%)	CART

AGE < 62 and HEAD ≤ 4 and ISS ≤ 25 Then Alive	648/822(78.8%)	CART

HEAD ≥ 5 and (Cg = 'No') and AGE < 22.85 Then Alive	60/73(82.2%)	CART

ISS ≥ 23 and AGE ≥ 80.50 Then Dead	45/55(81.8%)	CART

AGE < 61.70 and HEAD ≤ 4 and (MI = 'No') Then Alive	625/793(78.8%)	CART

AGE < 61.60 and (MI = 'No') and ISS < 42 and (Cg = 'Yes') and HEAD ≤ 3 Then Alive	47/56(83.9%)	CART

AGE < 61.60 and HEAD ≤ 4 and (MI = 'No') and (Cg = 'No') Then Alive	421/503(83.7%)	CART

AGE ≥ 61.60 and ISS ≥ 23 and (Cg = 'Yes') Then Dead	44/54(81.5%)	CART

AGE < 61.70 and HEAD ≤ 4 and (ISS < 27) Then Alive	646/820(78.8%)	CART

HEAD ≥ 5 and (Cg = 'No') and 22.35 ≤ AGE < 25.85 and (MI = 'No') and ISS ≥ 30 Then Dead	10/13(76.9%)	CART

ISS ≥ 23 and 61.70 ≤ AGE < 74.10 and EDRTS < 2.88 Then Dead	21/28(75.0%)	CART

ISS ≥ 23 and AGE ≥ 74.10 Then Dead	92/119(77.3%)	CART

(MI = 'No') and HEAD ≤ 4 and AGE ≤ 62 ISS ≤ 38 Then Alive	508/612(83%)	C4.5

3 < HEAD ≤ 4 and (MI = 'No') and (ID = 'Yes') and (Cg = 'No') and ISS ≤ 59 Then Alive	138/175(78.9%)	C4.5

HEAD > 1 and (MI = 'Yes') and ISS > 22 Then Dead	59/70(84.3%)	C4.5

HEAD > 3 and (MI = 'Yes') Then Dead	49/58(84.5%)	C4.5

(Cg = 'No') and (MI = 'No') and 2 < HEAD ≤ 4 and EDRTS ≤ 2.2 and (ARDS = 'Yes') and ISS ≤ 38 Then Dead	12/15(80%)	C4.5

(MI = 'No') and (ID = 'Yes') and (Cg = 'Yes') and AGE > 61.6 Then Dead	24/32(75%)	C4.5

(ID = 'No') and HEAD ≤ 3 and AGE ≤ 82.6 and ISS ≤ 22 Then Alive	236/305(77.4%)	C4.5

HEAD ≤ 4 and (MI = 'No') and AGE ≤ 60.8 and ISS ≤ 38 Then Alive	504/607(83%)	C4.5

HEAD ≤ 4 and AGE ≤ 78 and EDRTS > 7.55 and ISS ≤ 30 Then Alive	207/263(78.7%)	C4.5

(MI = 'Yes') and ISS > 27 Then Dead	50/60(83.3%)	C4.5

HEAD ≤ 3 and AGE ≤ 78 and 11 < ISS ≤ 27 Then Alive	290/368(78.8%)	C4.5

(Cg = 'No') and HEAD ≤ 3 and AGE ≤ 78 Then Alive	353/459(76.9%)	C4.5

(MI = 'Yes') and EDRTS ≤ 5.39 Then Dead	41/51(80.4%)	C4.5

HEAD > 0 and (MI = 'Yes') and THORAX > 2 and (ID = 'No') Then Dead	50/63(79.4%)	C4.5

(Cg = 'No') and (MI = 'No') and 2 < HEAD ≤ 4 and EDRTS ≤ 1.47 and (ARDS = 'Yes') and ISS ≤ 41 Then Dead	13/17(76.5%)	C4.5

HEAD ≤ 4 and (MI = 'No') and AGE ≤ 62 and ISS ≤ 41 Then Alive	555/678(81.9%)	C4.5

HEAD ≤ 4 and AGE ≤ 79.6 and EDRTS > 7.55 and ISS ≤ 30 Then Alive	214/275(77.8%)	C4.5

(MI = 'No') and HEAD ≤ 4 and AGE ≤ 61.6 and ISS ≤ 34 Then Alive	469/562(83.5%)	C4.5

HEAD ≤ 3 and AGE ≤ 61.6 and ISS ≤ 38 Then Alive	420/503(83.5%)	C4.5

(MI = 'Yes') and (ID = 'Yes') and AGE > 68.5 Then Dead	47/60(78.3%)	C4.5

(ARDS = 'No') and HEAD ≤ 3 and AGE ≤ 61.9 Then Alive	276/326(84.7%)	C4.5

(ID = 'No') and (MI = 'Yes') and EDRTS > 5.39 and ISS ≤ 14 Then Alive	21/25(84%)	C4.5

Though these rules are not reliable enough for practical use, they can contain pattern information which may be of interest to physicians. Cg stands for coagulopathy; MI for myocardial infarction; ARDS for Acute Respiratory Distress Syndrome; EDRTS for Emergency Department Revised Trauma Score; ISS for Injury Severity Score; ID for Insulin-Dependent.

**Table 12:** Extracted reliable rules for outcome prediction (> 85% accuracy)

Rules	Test Acc.	Method
HEAD ≤ 3 and AGE < 43.45 and FSBP < 143.50 and ISS ≤ 33 and EDRTS < 0.87 and THORAX ≥ 2 Then Rehab	17/19(89.5%)	CART

EDRTS < 5.36 and HEAD ≤ 3 and 33 ≤ FSBP ≤ 143 and ISS ≥ 33.50 Then Rehab	69/79(87.3%)	CART

HEAD ≥ 4 and FSBP < 171 and EDRTS < 2.25 Then Rehab	125/135(92.6%)	CART

2.25 ≤ EDRTS < 5.36 and HEAD ≥ 4 and FSBP < 171 and AGE ≥ 10.90 Then Rehab	45/52(86.5%)	CART

EDRTS ≥ 5.36 and AGE < 48.15 and THORAX ≥ 1 and ISS ≤ 21 Then Home	23/27(85.2%)	CART

EDRTS ≥ 5.36 and AGE ≥ 48.15 and EDGCSTOTAL ≥ 9 and ISS ≤ 25 Then Rehab	61/65(93.8%)	CART

EDRTS < 5.02 and HEAD ≤ 3 and 11.65 ≤ AGE < 24.40 and EDGCSTOTAL ≤ 8 and FSBP ≥ 108 and THORAX ≤ 4 Then Rehab	24/28(85.7%)	CART

EDRTS < 5.02 and HEAD ≤ 3 and 26.05 ≤ AGE < 37.30 and EDGCSTOTAL ≤ 8 and FSBP ≥ 108 Then Rehab	22/24(91.7%)	CART

EDRTS < 5.02 and HEAD ≤ 3 and AGE ≥ 43.30 Then Rehab	50/55(90.9%)	CART

EDRTS < 5.02 and HEAD ≥ 4 Then Rehab	179/201(89.1%)	CART

EDRTS ≥ 5.02 and AGE < 48.15 and THORAX ≥ 1 and ISS ≤ 21 Then Home	23/27(85.2%)	CART

EDRTS ≥ 5.02 and 24.25 ≤ AGE < 48.15 and THORAX ≥ 1 and 22 ≥ ISS < 28 and FSBP < 146 Then Home	11/12(91.7%)	CART

EDRTS ≥ 5.02 and AGE < 48.15 and THORAX ≥ 1 and ISS ≥ 22 and 74 ≤ FSBP ≤ 93 Then Rehab	10/11(90.9%)	CART

EDRTS ≥ 5.02 and AGE ≥ 48.15 and EDGCSTOTAL ≥ 9 and ISS ≥ 24.50 Then Rehab	104/122(85.2%)	CART

EDRTS < 2.69 and 113 ≤ FSBP ≤ 170 and HEAD ≤ 3 Then Rehab	49/57(86.0%)	CART

EDRTS < 2.69 and FSBP ≤ 170 and HEAD ≥ 4 Then Rehab	126/137(92.0%)	CART

EDRTS ≥ 2.69 and AGE < 45.10 and THORAX ≥ 1 and ISS ≤ 21 and EDGCSTOTAL ≥ 7 Then Home	24/28(85.7%)	CART

2.69 ≤ EDRTS < 5.02 and 22.80 ≤ AGE < 48.15 and THORAX ≥ 1 and ISS ≥ 22 and (ARDS = 'No') Then Rehab	25/28(89.3%)	CART

EDRTS ≥ 2.69 and 48.15 ≤ AGE < 84.30 and ISS ≥ 25 Then Rehab	74/79(93.7%)	CART

EDRTS < 5.02 and 39.25 ≤ AGE < 51.35 and HEAD ≤ 3 and FSBP ≤ 116 and ISS ≤ 33 Then Rehab	12/13(92.3%)	CART

EDRTS < 2.69 and AGE < 51.10 and HEAD ≥ 4 and FSBP < 179 Then Rehab	117/126(92.9%)	CART

2.69 ≤ EDRTS < 5.02 and 10.90 ≤ AGE < 51.10 and HEAD ≥ 4 and 82 ≤ FSBP < 179 and (ARDS = 'No') Then Rehab	28/31(90.3%)	CART

EDRTS < 5.02 and AGE ≥ 51.35 Then Rehab	48/52(92.3%)	CART

EDRTS ≥ 5.02 and AGE < 27.35 and THORAX < 1 and FSBP ≥ 123 and HEAD ≤ 4 Then Rehab	13/15(86.7%)	CART

EDRTS ≥ 5.02 and AGE < 48.15 and THORAX ≥ 1 and ISS ≤ 21 Then Home	23/27(85.2%)	CART

EDRTS ≥ 5.02 and AGE ≥ 48.15 and EDGCSTOTAL ≥ 7 and ISS ≥ 25 Then Rehab	61/66(92.4%)	CART

EDRTS < 5.02 and HEAD ≤ 2 and AGE ≥ 31.90 and ISS ≤ 39 and THORAX ≥ 2 Then Rehab	23/26(88.5%)	CART

EDRTS < 5.02 and HEAD ≥ 3 and AGE ≥ 23.15 Then Rehab	249/281(88.6%)	CART

EDRTS ≥ 5.02 and AGE ≥ 56.55 and EDGCSTOTAL ≥ 9 and ISS ≤ 24 and (ARDS = 'No') Then Rehab	33/34(97.1%)	CART

AGE < 24 and EDRTS < 0.58 and HEAD ≤ 3 and ISS ≤ 34 Then Rehab	19/20(95.0%)	CART

26.05 ≤ AGE < 47.15 and EDRTS < 4.75 and HEAD ≤ 3 and ISS ≤ 34 and THORAX ≥ 2 and EDGCSTOTAL ≤ 7 Then Rehab	36/41(87.8%)	CART

AGE < 48.15 and EDRTS < 4.75 and HEAD ≤ 3 and ISS ≥ 35 and EDGCSTOTAL ≤ 7 Then Rehab	18/20(90.0%)	CART

AGE < 48.15 and EDRTS < 2.69 and HEAD ≥ 4 Then Rehab	115/126(91.3%)	CART

23.15 ≤ AGE < 48.15 and EDRTS ≥ 4.75 and ISS ≥ 25 and HEAD ≤ 0 and FSBP ≥ 69 Then Rehab	17/19(89.5%)	CART

23.15 ≤ AGE < 26.75 and EDRTS ≥ 4.75 and ISS ≥ 25 and 1 ≤ HEAD ≤ 4 and FSBP ≥ 69 Then Rehab	11/11(100.0%)	CART

48.15 ≤ AGE < 85.70 and ISS ≥ 25 Then Rehab	122/133(91.7%)	CART

AGE < 48.15 and THORAX ≥ 1 and ISS ≥ 22 and EDRTS ≥ 5.36 and 73 ≤ FSBP < 120 Then Home	20/22(90.9%)	CART

EDRTS < 5.02 and HEAD ≤ 2 and AGE ≥ 30.25 Then Rehab	31/35(88.6%)	C4.5

EDRTS < 5.02 and HEAD ≤ 3 and ISS ≥ 15 Then Rehab	256/294(87.1%)	C4.5

EDRTS ≥ 5.02 and AGE ≤ 48.15 and EDGCSTOTAL ≥ 9 and ISS ≤ 24 and THORAX ≤ 3 and FSBP ≥ 93 Then Rehab	110/127(86.6%)	C4.5

EDRTS ≥ 2.69 and AGE < 26.75 and ISS ≥ 25 and HEAD ≥ 5 Then Rehab	33/37(89.2%)	C4.5

EDRTS ≥ 7.12 and 26.75 ≤ AGE < 43.25 and ISS ≥ 25 and 1 ≤ HEAD ≤ 2 Then Rehab	15/17(88.2%)	C4.5

EDRTS < 2.69 and HEAD ≤ 3 and AGE < 38.30 and 108 ≤ FSBP < 192 Then Rehab	38/43(88.4%)	C4.5

EDRTS < 2.69 and HEAD ≥ 4 Then Rehab	132/146(90.4%)	C4.5

EDRTS ≥ 2.69 and AGE < 48.15 and 84 ≤ FSBP ≤ 93 Then Rehab	18/21(85.7%)	C4.5

2.69 ≤ EDRTS < 4.75 and 11.65 ≤ AGE < 48.15 and FSBP ≥ 122 and (ARDS = 'No') Then Rehab	33/36(91.7%)	C4.5

EDRTS ≥ 2.69 and AGE ≥ 48.15 and ISS ≥ 26 Then Rehab	66/71(93.0%)	C4.5

EDGCSTOTAL ≤ 5 and ISS ≥ 15 and FSBP ≤ 177 and THORAX ≥ 4 Then Rehab	252/284(88.7%)	C4.5

EDGCSTOTAL ≥ 6 and AGE ≥ 48.15 and ISS ≥ 26 Then Rehab	66/72(91.7%)	C4.5

THORAX ≤ 2 and AGE ≤ 33.9 and EDRTS ≤ 5.03 Then Rehab	62/72(86.1%)	C4.5

(ID = 'Yes') and (Cg = 'No') Then Rehab	11/12 (91.7%)	C4.5

HEAD ≤ 0 and THORAX ≤ 1 and AGE ≤ 59.7 and ISS > 5 Then Rehab	28/32(87.5%)	C4.5

Reliable rules are defined as those with accuracy greater than 85%. FSBP represents initial blood pressure; ISS stands for Injury Severity Score; EDGCSTOTAL is the total Glasgow Coma Score recorded in the emergency department; EDRTS is the Emergency Department Revised Trauma Score; ARDS stands for Acute Respiratory Distress Syndrome.

**Table 13:** Extracted supporting rules for outcome prediction (75% – 85% accuracy)

Rules	Test Acc.	Method
EDRTS ≥ 5.36 and EDGCSTOTAL ≥ 9 and ISS ≤ 24 and THORAX ≤ 3 and AGE ≥ 53.95 and FSBP ≥ 93 Then Rehab	49/62(79.0%)	CART

EDRTS ≥ 7.12 and AGE < 47.55 and THORAX ≥ 1 and 28 ≤ ISS < 35 and 94 ≤ FSBP ≤ 135 Then Rehab	16/20(80.0%)	CART

EDRTS ≥ 2.69 and AGE < 22.80 and THORAX ≥ 1 and ISS ≥ 22 and 123 ≤ FSBP ≤ 139 Then Rehab	11/13(84.6%)	CART

EDRTS ≥ 7.70 and 22.80 ≤ AGE < 45.90 and THORAX ≥ 1 and ISS ≥ 28 and FSBP ≥ 76 Then Rehab	31/39(79.5%)	CART

5.02 ≤ EDRTS < 7.12 and AGE < 45.90 and THORAX ≥ 1 and 22 ≤ ISS ≤ 39 Then Rehab	9/12(75.0%)	CART

EDRTS ≥ 7.12 and AGE < 48.15 and ISS ≥ 25 and HEAD ≤ 4 and THORAX ≥ 1 and 69 ≤ FSBP < 98 Then Rehab	15/19(78.9%)	CART

EDRTS ≥ 2.69 and AGE < 47.80 and ISS ≤ 24 and HEAD ≤ 2 and Then Home	43/56(76.8%)	CART

2.69 ≤ EDRTS < 5.02 and 26.75 ≤ AGE < 47.80 and ISS ≥ 25 and HEAD ≥ 1 Then Rehab	28/34(82.4%)	CART

EDRTS ≥ 2.69 and ISS ≤ 24 and THORAX ≤ 3 and HEAD ≥ 3 Then Rehab	151/182(83%)	CART

EDRTS ≥ 4.75 and AGE < 48.15 and FSBP ≥ 94 and THORAX ≥ 1 and ISS ≤ 21 Then Home	21/25(84.0%)	CART

EDRTS ≥ 2.69 and AGE ≥ 48.15 and ISS ≤ 25 and THORAX ≤ 3 and FSBP ≥ 80 Then Rehab	59/74(79.7%)	CART

EDGCSTOTAL ≥ 7 and 26.75 ≤ AGE < 43.00 and ISS ≥ 25 and FSBP ≥ 138 and HEAD ≥ 3 Then Rehab	12/16(75.0%)	CART

EDGCSTOTAL ≥ 6 and AGE ≥ 50.60 and ISS ≤ 25 and THORAX ≥ 3 and HEAD ≤ 4 and FSBP ≥ 74 Then Rehab	61/79(77.2%)	CART

(ID = 'Yes') and AGE > 44 and (ARDS = 'Yes') Then Rehab	30/39(76.9%)	C4.5

THORAX ≤ 3 and ISS > 18 Then Rehab	342/431(79.4%)	C4.5

(Cg = 'No') and 18.4 < AGE ≤ 59.7 and ISS > 30 Then Rehab	162/199(81.4%)	C4.5

Though these rules are not reliable enough for practical use, they can contain pattern information which may be of interest to physicians. FSBP represents initial blood pressure; ISS stands for Injury Severity Score; EDGCSTOTAL is the total Glasgow Coma Score recorded in the emergency department; EDRTS is the Emergency Department Revised Trauma Score; ARDS stands for Acute Respiratory Distress Syndrome; Cg for coagulopathy.

**Table 14:** Extracted reliable rules for ICU days prediction (> 85% accuracy)

Rules	Test Acc.	Method
(AIRWAY = 'Need') and 115 ≤ ED-BP < 156 and AGE ≥ 47.05 and Then ICU stay days ≥ 3	14/15(93.3%)	CART

(AIRWAY = 'Need') and 115 ≤ ED-BP < 156 and ED-RESP < 18 and 4.35 ≤ AGE < 14.5 Then ICU stay days ≥ 3	12/12(100%)	CART

(AIRWAY = 'No Need') and ED-RESP ≥ 21 and 45 ≤ AGE < 55.85 Then ICU stay days ≤ 2	10/11(90.1%)	CART

(AIRWAY = 'Need') and ED-BP < 91 Then ICU stay days ≥ 3	14/14(100%)	CART

(AIRWAY = 'Need') and 93.5 ≤ ED-BP < 156.5 and ED-PULSE ≥ 60.5 and AGE ≥ 54.2 Then ICU stay days ≥ 3	10/10(100%)	CART

(AIRWAY = 'Need') and 94 ≤ ED-BP < 156 and ED-PULSE ≥ 61 and ED- RESP < 19 and 18.45 ≤ AGE < 44.5 Then ICU stay days ≥ 3	60/76(86.6%)	CART

(AIRWAY = 'No Need') and AGE < 52.9 and ED-BP ≥ 107 and ED- GCS ≥ 11 Then ICU stay days ≤ 2	175/192(91.1%)	CART

(AIRWAY = 'Need') and ED-BP < 150.5 and ED-RESP < 19 and AGE ≥ 4.9 and ED-PULSE ≥ 138 Then ICU stay days ≥ 3	18/20(90%)	CART

(AIRWAY = 'Need') and ED-RESP < 19 and ED-PULSE < 138 and ED- BP < 115 and 10.9 ≤ AGE < 47.3 Then ICU stay days ≥ 3	31/33 (93.9%)	CART

(AIRWAY = 'No Need') and AGE < 37.1 and ED-GCS ≥ 11 and ED- BP ≥ 125 Then ICU stay days ≤ 2	89/90(98.9%)	CART

(AIRWAY = 'No Need') and AGE < 37.1 and ED-GCS ≥ 11 and ED- BP < 119 Then ICU stay days ≤ 2	39/44(88.6%)	CART

(AIRWAY = 'No Need') and AGE < 37.1 and ED-GCS ≥ 13 and 119 ≤ ED- BP < 125 and ED-PULSE ≥ 90 Then ICU stay days ≤ 2	21/22(95.5%)	CART

(AIRWAY = 'Need') and 146 ≤ ED-BP < 156 and AGE < 22.5 Then ICU stay days ≥ 3	11/12(91.2%)	CART

(AIRWAY = 'No Need') and AGE < 37.05 and ED-GCS ≥ 9 Then ICU stay days ≤ 2	157/172(91.3%)	CART

(AIRWAY = 'No Need') and 37.05 ≤ AGE < 46.9 and ED-RESP < 21 and ED-PULSE < 121 Then ICU stay days ≤ 2	23/25(92%)	CART

(AIRWAY = 'No Need') and ED-RESP < 21 and ED-PULSE < 121 and AGE ≥ 49.7 and ED-BP ≥ 141 Then ICU stay days ≤ 2	12/13(92.3%)	CART

(AIRWAY = 'No Need') and AGE < 37.05 and ED-GCS < 10 and 114 ≤ ED- BP < 142 Then ICU stay days ≤ 2	11/12(91.7%)	CART

(AIRWAY = 'Need') and ED-BP < 91.5 Then ICU stay days ≥ 3	14/14(100%)	CART

(AIRWAY = 'Need') and 91 ≤ ED-BP < 156 and 95.5 ≤ ED-PULSE < 102.5 Then ICU stay days ≥ 3	15/17(88.2%)	CART

(AIRWAY = 'No Need') and AGE < 52.9 and ED-BP ≥ 99 and ED- GCS ≥ 13 Then ICU stay days ≤ 2	177/196(90.3%)	CART

(AIRWAY = 'No Need') and ED-BP < 134 and 37.05 ≤ AGE < 67.35 and ED-RESP < 19 Then ICU stay days ≤ 2	11/12(91.7%)	CART

(AIRWAY = 'Need') and ED-PULSE ≥ 62 and AGE ≥ 24.35 and ED- BP < 110 Then ICU stay days ≥ 3	26/29(89.7%)	CART

(AIRWAY = 'Need') and 110 ≤ ED-BP < 180 and ED-PULSE ≥ 62 and 47.05 ≤ AGE < 68.2 Then ICU stay days ≥ 3	16/17(94.1%)	CART

(AIRWAY = 'No Need') and AGE < 37 and ED-GCS ≥ 13 Then ICU stay days ≤ 2	147/159(92.5%)	CART

(AIRWAY = 'No Need') and AGE ≥ 37 and 135 ≤ ED-BP < 163 Then ICU stay days ≤ 2	26/29(89.7%)	CART

(AIRWAY = 'Need') and ED-PULSE ≥ 62 and AGE ≥ 24.35 and ED- BP < 110 Then ICU stay days ≥ 3	31/35(88.6%)	CART

(AIRWAY = 'Need') and ED-BP < 91 Then ICU stay days ≥ 3	14/14(100%)	CART

(AIRWAY = 'Need') and 93 ≤ ED-BP < 156 and ED-RESP < 19 and AGE ≤ 54.05 Then ICU stay days ≥ 3	10/10(100%)	CART

(AIRWAY = 'Need') and ED-RESP < 19 and 93 ≤ ED-BP < 119 and 18.45 ≤ AGE < 47.3 Then ICU stay days ≤ 3	24/26(92.3%)	CART

(AIRWAY = 'No Need') and ED-GCS ≥ 11 and ED-BP ≥ 88 and AGE < 37.05 Then ICU stay days ≤ 2	148/160(92.5%)	CART

Age ≤ 42 and (Airway = 'No Need') and ED-PULSE ≤ 137 and ED- RESP > 19 Then ICU stay days ≤ 2	100/116(86.2%)	C4.5

Age > 37 and ED-BP ≤ 95 Then ICU stay days ≥ 3	14/14(100%)	C4.5

Reliable rules are defined as those with accuracy greater than 85%. ED-BP is Emergency Department Blood Pressure; ED-RESP is Emergency Department Respiratory Rate; ED-PULSE is Emergency Department Pulse Rate; ED-GCS is Emergency Department Glasgow Coma Score.

**Table 15:** Extracted supporting rules for ICU days prediction (75% – 85% accuracy)

Rules	Test Acc.	Method
(AIRWAY = 'No Need') and ED-RESP < 21 and ED-BP < 142 ED- PULSE < 79 and 37.05 ≤ AGE < 44.15 Then ICU stay days ≤ 2	13/16(81.3%)	CART

(AIRWAY = 'No Need') and AGE ≥ 52.9 and ED-BP ≥ 141 Then ICU stay days ≤ 2	12/15(80%)	CART

(AIRWAY = 'Need') and 117 ≤ ED-BP < 135 and ED-RESP < 19 and 68 ≤ ED-PULSE < 138 and 15.05 ≤ AGE < 46.4 Then ICU stay days ≥ 3	23/28(82.1%)	CART

(AIRWAY = 'Need') and 136 ≤ ED-BP < 150 and ED-RESP < 19 and ED-PULSE < 138 and 15.05 ≤ AGE < 23.25 Then ICU stay days ≥ 3	10/13(77%)	CART

(AIRWAY = 'No Need') and 96 ≤ ED-BP < 163 and 39.15 ≤ AGE < 69.05 Then ICU stay days ≤ 2	44/55(80%)	CART

(AIRWAY = 'Need') and ED-BP < 156 and AGE ≥ 24.35 Then ICU stay days ≥ 3	76/96(79.2%)	CART

(AIRWAY = 'Need') and ED-BP < 146 and AGE < 17.85 and 135 ≤ ED-PULSE < 181 Then ICU stay days ≥ 3	9/11(81.2%)	CART

(AIRWAY = 'Need') and ED-BP < 146.5 and AGE < 17.85 and ED- PULSE < 131 and ED-RESP < 18 Then ICU stay days ≥ 3	15/20(75%)	CART

(AIRWAY = 'No Need') and ED-BP ≥ 141 Then ICU stay days ≤ 2	223/265(84.2%)	CART

(AIRWAY = 'Need') and ED-BP < 114 Then ICU stay days ≥ 3	44/52(84.6%)	CART

(AIRWAY = 'Need') and 114 ≤ ED-BP < 135.5 and ED-PULSE < 97 and 17.2 ≤ AGE < 46.95 and ED-RESP < 7 Then ICU stay days ≥ 3	10/13(77%)	CART

(AIRWAY = 'No Need') and AGE ≥ 52.9 and ED-BP ≥ 141 Then ICU stay days ≤ 2	12/15(80%)	CART

(AIRWAY = 'Need') and ED-BP < 114 Then ICU stay days ≥ 3	44/52(84.6%)	CART

(AIRWAY = 'Need') and 110.5 ≤ ED-BP < 180.5 and ED- PULSE ≥ 62 and 4.35 ≤ AGE < 44.5 and ED-GCS < 10 Then ICU stay days ≥ 3	35/46(76.1%)	CART

(AIRWAY = 'No Need') and AGE ≥ 37 and 135 ≤ ED-BP < 163 Then ICU stay days ≤ 2	31/35(88.6%)	CART

(AIRWAY = 'No Need') and 37.05 ≤ AGE < 55.6 and ED-GCS ≥ 11 and 88 ≤ ED-BP < 163 Then ICU stay days ≤ 2	40/49(81.6%)	CART

ED-BP > 100 and ED-RESP > 19 Then ICU stay days ≤ 2	145/180(80.6%)	C4.5

Though these rules are not reliable enough for practical use, they can contain pattern information which may be of interest to physicians. ED-BP is Emergency Department Blood Pressure; ED-RESP is Emergency Department Respiratory Rate; ED-PULSE is Emergency Department Pulse Rate; ED-GCS is Emergency Department Glasgow Coma Score.

## Discussion

We developed a computer-aided rule-base using significant variables selected via logistic regression, and showed that the approximations of the variables help increase rule quality. Our intent is to extract and formulate medical diagnostic knowledge into an appropriate set of transparent decision rules that can be used in a computer-assisted decision making system. The proposed method extracts the most significant variables using logistic regression with direct maximization likelihood estimation. By comparing the performances using five machine learning algorithms – AdaBoost, C4.5, CART, RBF neural network, and SVM – using all available variables and significant variables only, we found that using only the most significant variables provides a considerable improvement in performance. All five methods show improvement across all-available and significant-variables-only, indicating that our proposed selection method is robust and efficient.

The performance of individual rules was measured; reliable rules were identified as those with accuracy above 85%. In addition, all rules we selected were considered reliable if the number of cases in the dataset matching the rule was higher than a specified threshold. Rule sensitivity and specificity were also measured, and the average sensitivity and specificity for the three outcome pairs (alive/dead, home/read, severe/non-severe) are 87.4% and 88.4% respectively. This indicates that our method performs well. Some additional improvements may be needed to improve rule quality. In particular, large and well balanced datasets across all outcome classes could improve overall quality, as well as sensitivity and specificity. Full sensitivity and specificity results for the datasets are presented in Table 16.

**Table 16:** Rule sensitivity and specificity

	Off-site Dataset	Off-site Dataset	Helicopter Dataset
**Predictive Outcome**	**Alive/Dead**	**Home/Rehab**	**ICU stay Days**

**Sensitivity (> 85% rules)**	91.9%	88.7%	90.6%

**Specificity (> 85% rules)**	89.2%	87.7%	91%

**Sensitivity (75%–85% rules)**	86.2%	79%	82.5%

**Specificity (75%–85% rules)**	80.4%	80.1%	80.4%

One important issue in rule selection is how to deal with rules with accuracy below 85%. When using only the over-85% rules, some medical knowledge in the database might have been ignored. The accuracy of a rule may be low due to the lack of "database completeness", rather than a flaw in the rule itself. Therefore, rules with less than 85% accuracy cannot be completely removed from the rule based system. We will instead use those rules as additional "supporting rules" in suggesting possible treatment. For example, according to trauma experts, patients with a high ISS score (> 25) are least likely to survive. However, we found some rules with surprising implications. For instance, one of these "counterintuitive" rules pointed to the fact that there are 52 alive cases (3.3%) with ISS high scores (38). Of these 52 patients, 33 (63.5%) have high AIS head scores (≥ 4), and 38 patients (73%) are male. Considering the above conditions, surviving patients have lower thorax (average score = 2.61) and lower abdomen AIS scores (average score = 1.03) than fatal cases. These fatal cases typically have a higher head AIS score (average score = 5.08) than surviving patients (average head score = 3.90). In addition, we found that none of the surviving patients have complications such as coagulopathy, and only a few had a pre-existing disease (in particular, Insulin Dependency and Myocardial Infarction).

While only Acute Respiratory Distress Syndrome (ARDS) is usually considered an impact factor in predicted survival, according to the created rules, pre-existing conditions, Acute Respiratory Distress Syndrome (ARDS), Insulin Dependency, Myocardial Infarction, and Coagulopathy all have significant impact. Also, airway status (need/not need) was identified as a primary factor in predicting the number of ICU days for patients transported via helicopter.

Note that for ICU length of stay prediction, 74.6% of patients stayed at in ICU less than 2 days. Only 25.4% of patients stayed more than 2 days, and only 2.9% of those were in ICU for more than 20 days. This reinforces Eckhart's point that many patients are transported via helicopter unnecessarily. Therefore, the use of accurate ICU days prediction rules may help improve the efficiency of helicopter transport, considering cost effectiveness as well as the treatment of patients in critical condition.

## Conclusion

The results in this paper provide a framework to improve the physicians' diagnostic accuracy with the aid of machine learning algorithm. The resulting system is effective in predicting patient survival, and rehab/home outcome. A method has been introduced that creates a variety of reliable rules that make sense to physicians by combining CART and C4.5 and using only significant variables extracted via logistic regression. The resulting computer-aided decision-making system has significant benefits, both in providing rule-based recommendations and in enabling optimal resource utilization. This may ultimately assist physicians in providing the best possible care to their patients. The diagnosis of future patients may also be improved by analyzing all possible rules associated with their symptoms.

The system will be tested at all 17 hospitals of the Carolinas Healthcare System (CHS). Software that provides the computer-aided decision making system will be optimized and made available to the academic community as a web-based application, as well as a software tool on portable personal computing devices. Feedback from every hospital will then be considered and used to validate and improve the system.

## Competing interests

The authors declare that they have no competing interests.

## Authors' contributions

TH is responsible for obtaining the data and providing feedback on the medical impact of the results. SJ, RS and KN have designed the algorithms, analyzed the data, and drafted the manuscript. All authors have equal participation in the study as well as preparation of the final paper.

## Pre-publication history

The pre-publication history for this paper can be accessed here:

http://www.biomedcentral.com/1472-6947/9/2/prepub
